# Variations in Phytoestrogen Content between Different Mill Dates of the Same Diet Produces Significant Differences in the Time of Vaginal Opening in CD-1 Mice and F344 Rats but Not in CD Sprague-Dawley Rats

**DOI:** 10.1289/ehp.10165

**Published:** 2007-09-21

**Authors:** Julius E. Thigpen, Kenneth D.R. Setchell, Elizabeth Padilla-Banks, Joseph K. Haseman, Hannah E. Saunders, Gordon F. Caviness, Grace E. Kissling, Mary G. Grant, Diane B. Forsythe

**Affiliations:** 1 Comparative Medicine Branch, National Institute of Environmental Health Sciences, National Institutes of Health, Department of Health and Human Services, Research Triangle Park, North Carolina, USA; 2 Department of Pathology, Cincinnati Children’s Hospital Medical Center, Cincinnati, Ohio, USA; 3 Department of Pediatrics, University of Cincinnati College of Medicine, Cincinnati, Ohio, USA; 4 Biostatistics Branch, National Institute of Environmental Health Sciences, National Institutes of Health, Department of Health and Human Services, Research Triangle Park, North Carolina, USA

**Keywords:** dietary phytoestrogens, endocrine disruptors, rodent species/strain sensitivities in VO end points

## Abstract

**Background:**

The optimum test diet and rodent species/strain for evaluating endocrine-disrupting compounds (EDCs) are critical.

**Objectives:**

We conducted studies to evaluate rodent species sensitivity and the effects of diets varying in phytoestrogen content on the time of vaginal opening (VO) in CD-1 mice, Fischer 344 (F344) rats, and CD Sprague-Dawley (S-D) rats.

**Methods:**

Mice were weaned on postnatal day (PND) 15 and rats on PND19 and randomly assigned to control or test diets. Body weights, food consumption, and time of VO were recorded.

**Results:**

The time of VO was significantly advanced in F344 rats fed diets containing daidzein and genistein, whereas these same diets did not advance VO in S-D rats. When animals were fed the AIN-76A diet spiked with genistein, time of VO was significantly advanced at all doses in CD-1 mice, at the two highest doses in F344 rats, and at the highest dose in S-D rats. The time of VO in F344 rats was more highly correlated with the phytoestrogen content than with the total metabolizable energy (ME) of 12 diets.

**Conclusions:**

The S-D rat is less sensitive to dietary phytoestrogens compared with the F344 rat or the CD-1 mouse, suggesting that the S-D rat is not the ideal model for evaluating estrogenic activity of EDCs. The profound effects of dietary phytoestrogens on the time of VO, an estrogen-sensitive marker, indicate that a standardized open-formula phytoestrogen-free diet containing a low ME level should be used to optimize the sensitivity of estrogenic bioassays.

The use of rodent test diets containing up to 350 μg/g diet of total genistein equivalents (TGE) and various rat strains by the Organisation for Economical Co-operative Development (OECD) and the U.S. Environmental Protection Agency (EPA) for conducting uterotrophic assays is a subject of current concern (Kanno et al. 2001, 2003a; Owens et al. 2003; Thigpen et al. 2004a; U.S. EPA. 2003). The uterotrophic bioassay is used to evaluate the estrogenic activity of endocrine-disrupting compounds (EDCs). It is evident that different rodent species and strains differ substantially in their sensitivity to estrogens, and that the presence or absence of phytoestrogens in the diet and total metabolizable energy (ME) significantly influence the time of vaginal opening (VO) and uterine weight. The latter is the primary end point used in the uterotrophic bioassay.

We previously evaluated the influence of body weight gain and the total ME, dietary protein, fat, crude fiber, and phytoestrogen content of 20 different rodent diets on the outcome of uterotrophic bioassays in CD-1 mice (Thigpen et al. 2002). For diets with low phytoestrogen content, the increase in uterine weight was more highly correlated with the total dietary ME than with the phytoestrogen content. Diets with higher levels of total ME (3.5–3.8 Kcal/g diet) increased the rate of growth, resulting in increased body weight, increased endogenous estrogen levels, and earlier puberty and maturation. These effects reduce the sensitivity of the uterotrophic bioassay in immature CD-1 mice.

Total cumulative dietary energy intake has also been reported to determine the onset of puberty in female Wistar rats (Odum et al. 2004); consequently, ME is an important factor in the choice of diets for endocrine disruptor studies. In contrast, Odum et al. (2004) found that dietary phytoestrogen content of the diet(s) had little effect on the onset of puberty. Several other studies (Casanova et al. 1999; Kanno et al. 2001, 2003a, 2003b; Naciff et al. 2004; Owens et al. 2003; Wade et al. 2003; Yamasaki et al. 2002) have reported that dietary phytoestrogens have minimal impact, or do not influence the sensitivity of the uterotrophic bioassay, in Wistar or Sprague-Dawley (S-D) rats. Surprisingly, it was proposed by Owens et al. (2003) and adopted by the OECD that, in this bioassay, it is acceptable to routinely use rodent diets for research or regulatory purposes provided the levels of phytoestrogens are < 350 μg TGE/g diet in spite of earlier reports to the contrary (Kanno et al. 2002; Thigpen et al. 2004a; You et al. 2002). Casanova et al. (1999) evaluated the effects of soy and alfalfa-free diets and dietary genistein (200 and 1,000 μg/g diet) on sexual development in S-D rats. The time of VO was significantly advanced in rats fed only the 1,000 μg genistein/g diet. The authors did not suggest replacing soy- and alfalfa-based rodent diets with phytoestrogen-free diets in most developmental toxicology studies. However, they recommended phytoestrogren-free diets for endocrine toxicology studies at low doses to determine whether interactive effects may occur between dietary phytoestrogen and man-made chemicals.

In a series of studies we have shown that rodent diets significantly differ in phytoestrogen content and estrogenic activity and that these variations significantly influence the time of VO and/or uterine weight in CD-1 mice (Brown and Setchell 2001; Thigpen et al. 1987b, 1999, 2002). In our initial studies, when the results from all 20 diets were statistically analyzed as a group, the uterine weight was found to be more highly correlated with the ME, rather than with the phytoestrogen content of the 20 test diets evaluated in the study. Our results suggest that the use of diets containing low levels of total ME (3.0–3.1 Kcal/g diet) would increase the sensitivity of the uterotrophic bioassay and that more sensitive assays for determining the estrogenic activity of EDCs should be considered. In this context, Markey et al. (2001) reported that the VO assay was a more sensitive end point than the increase in uterine wet weight for determining the estrogenic activity of bisphenol A. This finding prompted us to evaluate the effects of 20 different rodent diets containing different concentrations of phytoestrogens on the time of VO in CD-1 mice (Thigpen et al. 2003, 2004b). In these later studies, we showed that the phytoestrogen content of the diet, daidzein and genistein (D&G), was more highly correlated with the time of VO in CD-1 mice than the total ME of the diet. Taken together, these results indicate that diets containing high concentrations of phytoestrogens or those having a high total ME significantly affect the results of the uterotrophic and VO bioassays. We also reported (Thigpen et al. 2003, 2004b) that the batch-to-batch variation in D&G content of different mill dates of the same PMI 5002 diet (Purina Mills Inc., St. Louis, MO) produces significant (*p* < 0.05) differences in the time of VO in CD-1 mice.

The objectives of the present studies were *a*) to determine whether the batch-to-batch variations in the phytoestrogen content between different mill dates of the same commercial diet (PMI 5002) would also produce significant differences in the time of VO in F344 and S-D rats; and *b*) to compare the sensitivity of the CD-1 mouse, the F344 rat, and the S-D rat to genistein added to the AIN-76A diet (Research Diets, Inc., New Brunswick, NJ) at concentrations of 0, 150, 300, or 450 μg/g diet. These studies were conducted to evaluate the importance of species and diet differences in measuring the estrogenic activity of endocrine disruptor compounds.

## Materials and Methods

### Experimental design

We conducted three separate studies. In study I, we determined the effects of the variations in the phytoestrogen content between different mill dates of the same PMI 5002 diet on the time of VO in F344 rats and S-D rats. In study II we used the time of VO to compare the differences in the sensitivity of three different rodents—the CD-1 mouse, the F344 rat, and the S-D rat—to purified genistein (99.3% pure, batch 2K030907; KingChem LLC, Allendale, NJ) added to an AIN-76A diet at 0, 150, 300, or 450 μg genistein/g diet. In study III we determined the effects of total ME and phytoestrogen content of 12 different rodent diets on the time of VO in F344 rats. The time of VO was established when the vagina exhibited complete canalization and patency, as previously described (Thigpen et al. 2003).

#### Animal housing

All studies were approved by the National Institute of Environmental Health Sciences (NIEHS) Animal Care and Use Committee. In all three studies, certified virus- and pathogen-free CD-1 mouse dams, F344 dams, and S-D dams (Charles River Laboratories, Raleigh, NC) with standardized litters, 10 female pups born on the same day, were received when the pups were 8 days old. Each dam and her pups were housed in a polypropylene cage containing autoclaved hardwood bedding (P.J. Murphy Forest Products Corp., Montville, NJ) in an animal facility accredited by the Association for Assessment and Accreditation of Laboratory Animal Care (AAALAC), International. Room conditions were as follows: room temperature, 72 ± 2°F, relative humidity, 40–60%; and 12-hr light–dark cycles. On arrival, the dams were fed the Zeigler soy/alfalfa-free phytoestrogen-reduced diet-II (Zeigler Bros. Inc., Gardners, PA) containing < 10 μg D&G/g diet and given reverse osmosis/deionized water *ad libitum* until the pups were weaned. All animals were observed daily and treated humanely with regard for alleviation of pain and suffering.

#### Study I design

In study I, we weaned F344 and S-D pups on postnatal day (PND) 19 and randomly assigned 4 pups/cage (16–20 pups/diet). Cages were randomly assigned to one of three different mill dates of the PMI 5002 test diet that naturally contained 98, 223, or 431 μg D&G/g diet, a diet with a low ME level (3.1 Kcal/g diet), or a control PMI 5K96 phytoestrogen-reduced diet (< 10 μg D&G/g diet; Purina Mills Inc.) of similar ME levels (3.15 Kcal/g diet). We observed weaned pups daily from PND19 until the time of VO and recorded body weights at weaning, at time of VO, and at weekly intervals thereafter until PND40. We measured food consumption by S-D rats (not F344 rats) at weekly intervals by weighing the food daily before and after each feeding period. All animal procedures complied with NIEHS/National Institutes of Health animal care guidelines and were approved by the NIEHS Animal Care and Use Committee. In all studies, animals were sacrificed by CO_2_ asphyxiation; we collected plasma in the morning (between 0900 hours and 1200 hours) in order from the lowest dose group to the highest. We obtained plasma from five S-D rats from each group on PND40 for measurement of total isoflavones. We did not collect plasma from F344 rats.

#### Study II design

In study II, we determined the effects of the AIN-76A diet, which naturally contains high ME levels of 3.83 Kcal/g diet (D11520; Research Diets, Inc.), spiked with genistein at 0, 150, 300, or 450 μg genistein/g diet on the time of VO in CD-1 mice, F344 rats, and S-D rats. We randomly assigned F344 rats and S-D rats to cages, as described above, and fed the control or test diets spiked with different levels of genistein. We observed pups daily from PND19 to PND40 to determine the time of VO, and we recorded body weights at weaning, time of VO, and weekly intervals until PND40. We also measured daily food consumption for F344 rats and S-D rats. We weaned CD-1 mouse pups on PND15 and randomly assigned five mice per cage (three cages per group). We randomly assigned cages to the control or test diets spiked with genistein. We determined the time of VO by observing animals daily from PND19 to PND30 and recorded body weights at weaning (PND15), at time of VO, and on PND22 and PND29. We collected plasma on PND30 from five mice from each group, and samples were assayed for genistein concentrations (Brown and Setchell 2001).

#### Study III design

In study III, we fed 12 different diets to F344 rats to compare the relative contribution of the phytoestrogen content and total ME of the diet to the time of VO. We recorded body weights as described above and fed the following diets: PMI 5K96 (an irradiated casein based diet; < 10 μg D&G/g diet); PMI 5001 maintenance diet; three different mill dates of the PMI 5002 certified diet; the Zeigler soy- and alfalfa-free phytoestrogen-reduced diet II (5412–01; Zeigler Brothers Inc., Gardners, PA); Harlan Teklad Global soy- and alfalfa-free diets 2014S, 2016S, and 2019S; Harlan Teklad 8656 (Harlan Teklad, Madison, WI); the AIN-76A casein-based diet (D11520); and the AIN-76A soy protein diet (Research Diets, Inc.).

### Assays for dietary phytoestrogens

We used a reverse-phase, high-performance liquid chromatography procedure (Brown and Setchell 2001; Setchell and Cole 2003) to assay the diets for the phytoestrogens daidzein, G, and their conjugates; formononetin; biochanin A; and coumestrol. We analyzed samples (5 g) of each diet in duplicate and in a “blinded” manner; the concentration of each phytoestrogen was expressed as aglycon equivalents in micrograms per gram (parts per million) of the diet (Brown and Setchell 2001; Setchell and Cole 2003). The between-batch precision of the method, expressed as coefficient of variation determined from replicate analyses (*n* = 16), ranged from 5.4 to 6.1% for total isoflavones in two feed samples having concentrations of 500 and 24,000 μg/g, respectively.

### Assays for plasma phytoestrogen

To correlate the total ME and phytoestrogen content in the diet with the time of VO and with the plasma isoflavone concentrations, we measured plasma total and individual isoflavone concentrations using stable-isotope dilution analysis with selected ion monitoring gas chromatography-mass spectrometry, as described previously (Brown and Setchell 2001). The concentrations of genistein, daidzein, and the intestinal bacterial metabolites equol and O-desmethyl-angolensin were quantified.

### Assays for mycotoxins and pesticides

The diets were assayed for the mycotoxins/aflatoxins B_1_, B_2_, G_1_, and G_2_; ochratoxin A; trichothecene (T-2); zearalenone; and deoxy-nivalenol at an independent laboratory (Romer Labs, Inc., Union, MO). The diets were assayed for > 30 pesticide residues, including organochlorines, organophosphates, carbamates, and chlorinated hydrocarbons, by an independent laboratory (Lancaster Laboratories, Inc., Lancaster, PA).

### Statistical method

We analyzed data from five separate experiments involving a total of 15 diets and 160 CD-1 mice, 383 F344 rats, and 160 S-D rats. Multiple regression procedures were used to determine whether the phytoestrogen and/or ME content of a diet was predictive of the time point at which 50% of the animals showed complete VO. These analyses also evaluated the impact of experiment-to-experiment variability. All possible pair-wise comparisons among the diets were made by using Fisher’s least significant difference (LSD) test (Snedecor and Cochran 1980). We also used Fisher’s LSD test to evaluate differences in body weight at weaning (PND15 for mice and PND19 for rats), at time of VO, and at weekly intervals.

## Results

### Study I: F344 rats versus S-D rats fed different mill dates of the PMI 5002 diet

**Figure 1:**
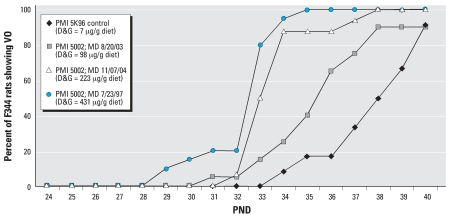
The effect of batch-to batch variation in the total D&G content in different mill dates (MD) of the same PMI 5002 diet on the timing of VO in F344 rats.

**Figure 2:**
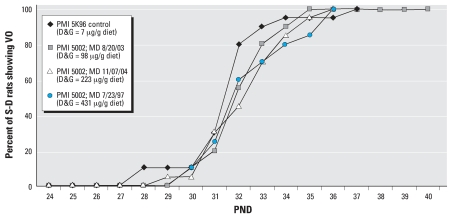
The effect of batch-to batch variation in the total D&G content in different mill dates (MD) of the same PMI 5002 diet on the timing of VO in S-D rats.

The effect of variations in the phytoestrogen content of different mill dates of the same PMI 5002 diet on the timing of VO in F344 rats and S-D rats are shown in Figures 1 and 2, respectively.

The average time of VO was significantly advanced in F344 rats fed diets with the highest phytoestrogen content. The mean ± SE time of VO occurred on PND35.5 ± 3.8, PND33.9 ± 1.5, and 32.6 ± 1.5 days (*p* < 0.05) with diets containing 98, 223, and 431 μg D&G/g diet, respectively. However, the mean time of VO for the control group fed a PMI 5K96 casein-based diet that contained only trace levels of phytoestrogens (7 μg D&G/g) was 38.1 ± 2.8 days (Figure 1). In contrast, for S-D rats, the mean time of VO was not significantly different among the three different mill dates of the PMI 5002 diet (32.5 ± 1.4, 32.7 ± 1.9, and 32.7 ± 1.9 days for PMI diets containing 98, 223, and 431 D&G/g diet, respectively). For comparison, the average time of VO for the control S-D rats was 31.9 ± 1.9 days when fed the PMI 5K96 diet (Figure 2). When comparing S-D rats with F344 rats, the difference in the mean time of VO was 6.2 days with the phytoestrogen-free low ME control PMI 5K96 diet.

**Figure 3:**
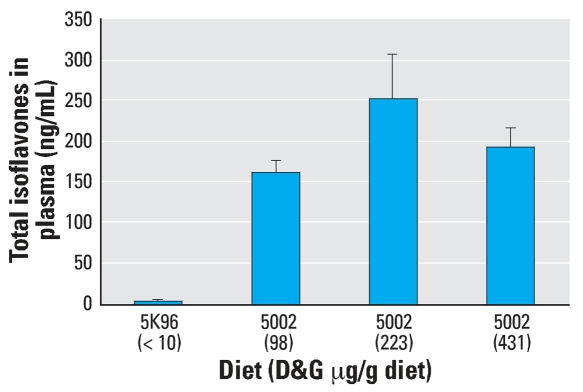
Effect of batch-to batch variation in total D&G content in different mill dates of the same PMI 5002 diet on the concentration of total isoflavones (mean ± SE) in the plasma of S-D rats.

Plasma total isoflavone concentrations on PND40 in S-D rats fed these diets are shown in Figure 3. Only trace levels of phytoestrogens were detected in the plasma of S-D rats fed the control PMI 5K96 diet. In contrast, high concentrations of phytoestrogens were detected in the plasma of S-D rats fed the three different mill dates of the PMI 5002 diet containing 98, 223, or 431 μg D&G/g diet.

We observed no significant differences in body weights at weaning between the different groups of F344 rats fed the natural ingredient diet (PMI 5K96) or the three different mill dates of the PMI 5002 diet. Similarly, there were no significant differences in body weights at weaning within the different groups of S-D rats fed the PMI natural ingredient diets (Table 1).

Body weights, daily food consumption, and the estimated dose of D&G for S-D rats at each time point are shown in Table 2. S-D rats consumed an estimated dose of approximately 60 mg/kg/day without advancing the time of VO. No significant differences in food consumption were observed within the groups of S-D rats fed the different mill dates of the PMI 5002 diet or the PMI 5K96 control diet.

### Study II: comparative sensitivity to genistein

**Figure 4:**
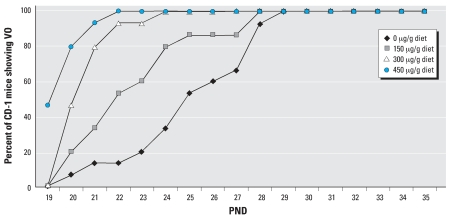
Effect of AIN-76A diet spiked with 0, 150, 300, or 450 μg genistein/g diet on the time of VO in CD-1 mice.

**Figure 5:**
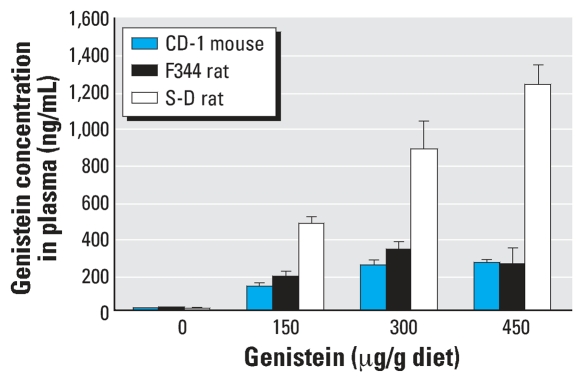
Mean concentration of genistein (± SE) in the plasma of CD-1 mice, F344 rats, and S-D rats fed the AIN-76A diet spiked with 0, 150, 300, and 450 μg genistein/g diet.

The effects of dietary genistein on the time of VO in CD-1 mice are shown in Figure 4. The time of VO was significantly advanced in CD-1 mice fed the AIN-76A diet containing 150, 300, or 450 μg genistein/g diet dose compared with the control AIN-76A genistein-free diet. Figure 5 shows the plasma genistein concentration of mice fed these diets compared with that of F344 and S-D rats. Only trace levels of genistein were detected in the plasma of mice fed the negative control diet, whereas high levels of genistein were detected in all plasma samples from mice fed the genistein-spiked diets.

At weaning (PND15), PND22, or PND30, we found no significant differences in body weight between the groups of CD-1 mice fed the control or genistein test diets. However, significant differences were observed in body weights on the day of VO in CD-1 mice exposed to genistein (Table 3). This body weight difference reflected primarily the earlier age at the time of VO in mice fed diets containing different levels of genistein.

**Figure 6:**
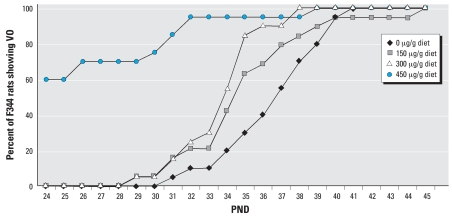
Effect of AIN-76A diet spiked with 0, 150, 300, or 450 μg genistein/g diet on the time of VO in F344 rats.

**Figure 7:**
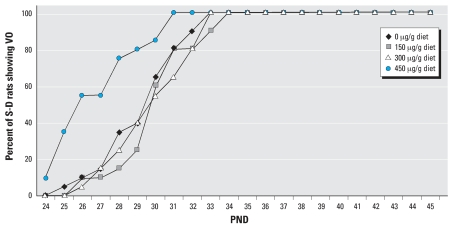
Effect of AIN-76A diet spiked with 0, 150, 300 or 450 μg genistein/g diet on the time of VO in S-D rats.

The mean time of VO in F344 rats and S-D rats fed different levels of genistein are shown in Figures 6 and 7, respectively. In F344 rats the time of VO was significantly (*p* < 0.05) earlier in animals exposed to 300 and 450 μg genistein/g diets than in those fed the control AIN-76A diet (Figure 6). For S-D rats fed the same diets, we observed significant (*p* < 0.05) advancement in the time of VO only with the diet containing 450 μg genistein/g diet (Figure 7). For comparison, the difference in mean time of VO was 7.2 days between S-D and F344 rats fed the phytoestrogen-free high ME control AIN-76A diet.

Body weights, daily feed consumption, and estimated dose of genistein for S-D and F344 rats fed the AIN-76A diet containing 0, 150, 300, or 450 μg genistein/g diet are shown in Table 4. From PND19 to PND26, F344 rats consumed more genistein than S-D rats. However, from PND26 to PND33 S-D rats consumed more genistein than F344 rats. We found no differences in the dose levels between F344 and S-D rats on PNDs 33–40.

**Figure 8:**
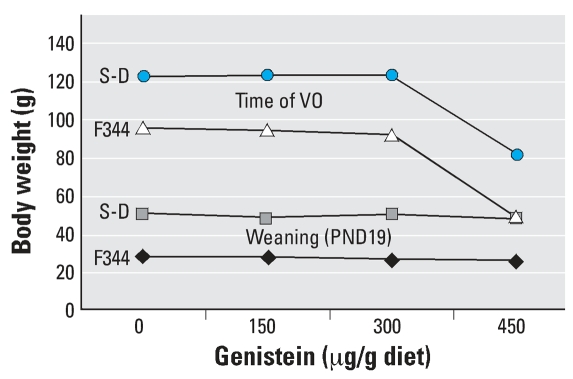
Effect of AIN-76A diet spiked with 0, 150, 300, or 450 μg genistein/g diet on the body weights of F344 rats versus S-D rats at weaning and at the time of VO.

In animals fed the AIN-76 diet spiked with 150, 300, or 450 μg genistein/g diet, plasma isoflavone concentrations in S-D rats were 2- to 5-fold higher than in F344 rats and CD-1 mice (Figure 5). In study II, we observed no significant differences in body weights within the different groups of F344 rats, or within the different groups of S-D rats at any stage of postnatal development (Table 1). As in study I, at all PND time points body weights were significantly (*p* < 0.05) lower in F344 rats than in S-D rats (Figure 8).

### Study III: effects of ME and D&G content of 12 different diets on time of VO in F344 rats

**Figure 9:**
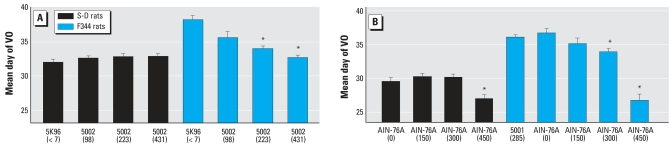
Comparison of VO times between S-D rats and F344 rats fed diets containing different concentrations of D&G or genistein. (*A*) Time of VO (mean ± SE) in S-D rats and F344 rats fed the control diet (5K96) or three different batches of the PMI 5002 natural ingredient diet containing different concentrations of D&G (< 7, 98, 223, and 431 μg/g diet) with similar ME levels. (*B*) Time of VO in S-D rats and F344 rats fed purified diets with high ME levels spiked with genistein at 0 (control), 150, 300, or 450 μg/g diet. *Significantly different at *p* < 0.05.

Table 5 shows the effects of 12 different diets with varying phytoestrogen content and total ME on the time of VO in F344 rats. The mean time of VO in F344 rats varied approximately 10 days (32.3–42.2 days) depending on the diet. Multiple regression analyses revealed that both phytoestrogen content and total ME were significantly (*p* < 0.01) correlated with the time of VO in F344 rats. Of the two variables, phytoestrogen content was the stronger predictor in the sense of being more significant statistically in the multiple regression (see Table 5 for more details). Figure 9A shows the comparison of mean time of VO in F344 rats and S-D rats weaned on PND19 and fed the PMI 5K96 control diet or three different mill dates of the PMI 5002 diet containing 98, 223, or 431 μg D&G/g diet. The mean time of VO varied up to 6 days (days 32–38) for F344 rats and only 1 day (days 32–33) for S-D rats. The mean time of VO in F344 rats and that of S-D rats, both weaned on PND19 and fed the AIN-76A diet spiked with 0, 150, 300, or 450 μg genistein/g diet, are shown in Figure 9B. With the presence of genistein in the diet, the mean time of VO varied approximately 10 days (days 27–37) in F344 rats and only 3 days (days 27–30) in S-D rats. Thus, diets with a high phytoestrogen content significantly advanced the time of VO in CD-1 mice and F344 rats, but less so in S-D rats (Figures 1, 4, and 6; Tables 3 and 5). These data confirm that the time of VO in rats is influenced by dietary phytoestrogen content and total ME; furthermore, different animal species and strains respond differently to these variables (Figure 9, Tables 4 and 5).

The difference in the mean time of VO between F344 rats and S-D rats due to strain, dietary phytoestrogen content, and total ME are summarized in Table 6. The mean time of VO varied 6.2–7.2 days due to the rat strain in animals fed the phytoestrogen-free control diets. The mean time of VO in F344 rats varied 5.5 days due to dietary phytoestrogens. In contrast, dietary phytoestrogens were responsible for a variation of only 0.8 days in the mean time of VO in S-D rats. The effects of ME on the mean time of VO was about the same for both rat strains (Table 6).

## Discussion

Commercial rodent diets are a major source of inadvertent estrogen exposure for laboratory animals (Brown and Setchell 2001; Thigpen et al. 1987a, 1992, 1998, 1999, 2003, 2004a). There are many potential sources of estrogenic substances in the diet, such as mycotoxins and pesticide residues. However, it is phytoestrogens that are quantitatively the major source of “estrogen” exposure for rodents. Phytoestrogens include primarily the soy bean isoflavones and coumestans derived from alfalfa, and these compound classes can profoundly influence the results of endocrine-sensitive end points (Boettger-Tong et al. 1998; Brown and Setchell 2001). Dietary phytoestrogens are biologically active, possessing many hormonal and non-hormonal properties, and consequently at significant levels of intake can affect growth and development, reproduction, gene expression, and sensitivity to carcinogens. Furthermore, the sensitivity of animals to phytoestrogens is also a function of species and strain, age, sex, dosage, route of administration, and duration of exposure (Ashby et al. 2000; Odum et al. 2001; Owens et al. 2003; Padilla-Banks et al. 2001; Spearow et al. 1999; Thigpen et al. 2004a; U.S. EPA. 2003; Yang and Bittner 2002).

Typical exposure level of rodents to levels of isoflavones contained in most commercial diets that are formulated with soy meal range from 80 to 160 mg/kg body weight/day which is far in excess of what is typically consumed when people eat soy foods on a daily basis (0.5–1.0 mg/kg body weight/day). However, there is wide variation in the phytoestrogen content of commercial rodent diets (Thigpen et al. 2004a, 2004b) because purified soy proteins can vary in phytoestrogen content by 3- to 4-fold (Setchell and Cole 2003), given the large natural variation in the isoflavone content of soybeans (Eldridge and Kwolek 1983; Hoeck et al. 2000; Hou and Chang 2002; Njitu et al. 1999; Simonne et al. 2000; Tsukamoto et al. 1995). Such variation poses a major problem in manufacturing diets with consistent compositions. It is possible to control the soy protein content of the diets, but it is extremely difficult to control or standardize the phytoestrogen content because soy proteins used by industry vary by less than 3% from batch to batch (Setchell and Cole 2003). Consequently, investigators using rodent diets formulated with soy meal face the prospect of being unable to control the extent of exposure to phytoestrogens; this may affect the results of estrogenic studies and make it difficult to both reproduce and compare results within or between laboratories.

The obvious solution to eliminating batch-to-batch variability in phytoestrogen content is to eliminate significant known sources of phytoestrogens by the removal of soybean meal or soy protein from commercial rodent diets used in studies that can be affected by dietary phytoestrogens. Soy protein or casein stripped of isoflavones and other estrogens by alcohol washing could possibly offer an alternative source of protein. The removal of soybean meal from all rodent diets is another potential option. Such a move would result in standardization of experimental results and an improvement in the sensitivity of bioassays for estrogenic substances. The results presented here reinforce the need to move in this direction and magnify the importance and need to use soy/alfalfa-free diets when conducting studies evaluating hormonal end points that can be affected by phytoestrogens (Brown and Setchell 2001; Thigpen et al. 1998, 1999, 2003, 2004a). At a minimum, the phytoestrogen content of the diet should be reported.

Results of the present study clearly demonstrate that the 3- to 4-fold variability in phytoestrogen content between different mill dates of the same diet produce statistically significant differences in the time of VO in CD-1 mice and F344 rats but not in S-D rats. Furthermore, our results show that the S-D rat is less sensitive to dietary phytoestrogens than either the CD-1 mouse or the F344 rat. Our findings that the dynamic response window or range for the mean time of VO for the three rodent strains fed different diets varied ∼ 10 days for the CD-1 mice (PNDs 20–30), and F344 rats (PNDs 32–42) and only ∼3 days for the S-D rats (PNDs 29–32) provides additional evidence that the S-D rat is less sensitive to dietary phytoestrogens and is not the most sensitive strain for conducting VO bioassays. This observation has significant implications for the selection of the most appropriate rodent species and strain to be used in testing for EDCs. Using three different batches of the PMI 5002 diet, we found an inverse relationship between the time of VO and the dietary phytoestrogen content for F344 rats. Mean VO times ranged from 32.6 days (431 μg/g diet) to 35.5 days (98 μg/g diet) for diets containing phytoestrogens; this was significantly earlier than the mean time of VO of 38.2 days when this strain was fed a diet essentially devoid of phytoestrogens. For S-D rats, the same batches of PMI 5002 diets resulted in VO times (32.4–32.7 days) that were no different from the control PMI 5K96 diet (31.8 days), even though plasma isoflavone concentrations in S-D rats fed different mill dates of the PMI 5002 diet were much higher than the total isoflavone plasma levels in S-D rats fed the PMI 5K96 control diet (Figure 3). The much higher plasma concentrations in the S-D rat versus the F344 rat and the CD-1 mouse may simply reflect the higher rate of food intake by this strain (Figure 5).

You et al. (2002) reported that an acceleration in the time of VO in exposed female offspring was the only observed effect of dietary genistein at 300 ppm (micrograms per gram of diet) or approximately 30–39 mg/kg/day. This difference in the S-D rats’ response to VO is probably caused by the difference in design of our study and that of You et al. (2002). In their study dams were fed the test diet (300 ppm) during gestation and weaning, and the female offspring were maintained on the test diet until VO was recorded. In our study, the dams, with their 8-day-old female pups, were placed on a phytoestrogen-free diet. After weaning (PND19), pups were placed on different mill dates of the same PMI 5002 test diet or on the control PMI 5K96 diet.

Strain differences in estrogen sensitivity were further evident from studies in which the AIN-76A diet (with a high ME level) was spiked with 0, 150, 300, or 450 μg/g diet of pure genistein. The F344 rats in study II showed significant differences in the time of VO in animals fed the diets with the two highest doses of genistein. A slight effect was also observed with the diet spiked with the lowest dose of genistein. This study showed that the mean time of VO in F344 rats varied from 36.75 to 26.75 days with increasing levels of genistein in the diet. On the other hand, the mean time of VO in S-D rats was only advanced by the diet containing the highest dose of genistein (450 μg/g diet). In contrast, F344 rats showed an advanced time of VO when fed the 300- or 450-μg genistein/g diet. S-D rats consumed more food per day than F344 rats, but surprisingly, based on the estimated dose of genistein, the F344 rats received more genistein from PND19 to PND26. However, this was reversed from PND26 to PND33 when S-D rats consumed a higher dose of genistein than F344 rats (Table 4).

When we looked at the plasma concentration of genistein for the CD-1 mouse and F344 and S-D rats (Figure 5), we found a much higher dose-related response of genistein concentration in the plasma of S-D rats compared with CD-1 mice and F344 rats. A possible explanation for the marked difference in response to the different diets between S-D rats and F344 rats or CD-1 mice is the apparent inability of S-D rats to efficiently metabolize genistein. This idea is supported by Helton et al. (1977), who suggested that isolated intact liver parenchymal cells from S-D rats were less efficient than the C3H mouse cells in their ability to covert 17α-ethynyl-estradiol into its metabolites. The slower clearance of genistein by female S-D rats was also reported by Sfakianos et al. (1997); their data showed that genistein is metabolized by the liver and absorbed by the intestinal wall, but a small amount appears in the urine. Genetic differences in the inability of S-D rats to metabolize isoflavones at the same rate as CD-1 mice and F344 rats may be contributing to the higher plasma level of free genistein. The plasma concentration in the S-D rat exposed to the PMI 5002 diet in study I shows a similar pattern of response to D&G in the diet, although this data could not be compared to plasma from the F344 rat or the CD-1 mouse.

Study III was designed to discern the relative role of ME versus phytoestrogen content in influencing the time of VO. When the diet had an ME in the 3.04–3.2 Kcal/g range, differences in the phytoestrogen content of the diet influenced the time of VO in F344 rats (i.e., diets with higher phytoestrogen contents resulted in consistently earlier VO times in this rat strain). However, data from study I for the four diets with comparable ME (3.10–3.15 Kcal/g) indicated that S-D rats showed no difference in time of VO, even though the phytoestrogen contents of the diets were significantly different. Again, these results indicate the relative insensitivity of the S-D rat to dietary phytoestrogens compared with the CD-1 mouse and the F344 rat.

The high variability in phytoestrogen content of commercial diets, evident from the differences in total isoflavone content of the three different mill dates of the same PMI diets tested, means that it would be difficult to obtain reproducible results in hormonal studies between different laboratories and, for that matter, within the same laboratory over time. Currently, few diets are certified for the phytoestrogen content. Compounding the problem is the fact that different rodent species or strains show differing responses to different diets. Using the least sensitive rodent strain or the wrong diet may lead to inaccurate results when assessing the estrogenicity of a substance. As early as 1987, we (Thigpen et al. 1987a) reported that rodent diets significantly differ in estrogenic activity and concluded that a “standardized diet” with minimal estrogenic activity should be used when comparing the effects of estrogenic compounds. Our findings presented here and earlier (Thigpen et al. 2003, 2004a) establish the importance of using a standardized phytoestrogen-free diet with low ME levels (3.0–3.1 Kcal/g diet) for the VO and uterotrophic bioassays to enhance the sensitivity of the assay.

Another critical factor to consider when selecting rodent species/strain for conducting VO bioassays is the variation in baseline data in the mean time of VO when animals are fed different diets with variable levels of phytoestrogens and ME. For example, our data confirm that the F344 rat reached puberty later than the S-D rat. The difference in the mean time of VO between F344 rats and S-D rats was 6.2 days when animals were fed the PMI 5K96 low-ME control diet and approximately 7.2 days when they were fed the AIN-76A high-ME control diet. In study III, the variation in the mean time of VO in F344 rats fed 12 different diets was approximately 10 days. This wider dynamic response window in the mean time of VO in the F344 rat suggests that using this model provides a greater opportunity to detect a weak estrogenic response to weak-acting EDCs than does the S-D rat.

Differing opinions exist regarding the choice of optimal diet and rodent strain for the uterotrophic bioassay used in the OECD program designed to evaluate the estrogenic activity of approximately 87,000 potential EDCs (Kanno et al. 2003b; Owens et al. 2003). The rat and mouse have been routinely used in uterotrophic bioassays for years. The OECD validation studies were performed primarily using S-D or Wistar rats. This was based on an understanding that both species are expected to be equivalent, and therefore one species should be acceptable for the worldwide validation in order to save time and money. The OECD proposed that it was acceptable to use diets with up to 350 μg/g TGE in this testing program when conducting uterotrophic assays in ovariectomized or immature S-D or Wistar rats (Kanno et al. 2003b; Owens et al. 2003). The OECD guidelines (OECD 2006) state that in some cases mice may be used instead of rats. Thus, modification of the protocol may be necessary for mice because the food consumption of mice on a body weight basis is higher than that of rats. Therefore, the phytoestrogen content of the diet should be lower for mice than for rats (Owens et al. 2003; Thigpen et al. 2002, 2003, 2004b). We have shown that the S-D rat is clearly less sensitive to estrogens. It is difficult to comprehend why an important testing program such as the OECD would adopt an assay using an animal species that is relatively insensitive at detecting estrogen activity, and at the same time would compound the problem by using a test diet with a significant level (< 350 μg/TGE) of background phytoestrogens. The diet seems especially problematic because of the wide batch-to-batch variability in the phytoestrogen content of rodent diets and the fact that vendors of these diets presently do not assay for known dietary estrogen and the phytoestrogens. Establishing threshold values for immature and adult mice and rats or setting limits, even < 350 μg/g TGE, for phytoestrogen content in the diet is not feasible given that it is impossible to manufacture diets that have constant levels of phytoestrogens. Any program designed to determine the estrogenicity of a chemical should use the most sensitive assay possible. This is an important consideration because some EDCs may have very long half-lives and thus, even with low-exposure levels, these compounds could accumulate in tissues. Our data on timing of the onset of VO confirms that the S-D rat is less sensitive to phytoestrogens than either the CD-1 mouse or the F344 rat. Therefore, it seems logical that one of the latter two strains would be a more appropriate model to ensure a more sensitive VO or uterotrophic assay. Although the proposed OECD acceptable dietary level of TGE (350 μg/g diet) (OECD 2006) may not have profound effects on uterine growth in the S-D rat, there is sufficient published data to show that phytoestrogens do have other genomic and nongenomic effects in this species (Brown and Setchell 2001). Furthermore, the use of diets containing phytoestrogens would not be appropriate for other strains of rats or for mice. In the VO or uterotrophic assays it is imperative that the test rodents be fed a standardized diet essentially devoid of phytoestrogens or one that has extremely low levels, because this will maximize the sensitivity of the assay. Based on our findings, we have suggested that the diet should contain no more than 20 μg/g TGE (Thigpen et al. 2004b), which is approximately at the detection limit (10 μg/g diet) of most HPLC assays for phytoestrogens. Additionally, the diet should ideally have a low level of ME, approximately 3.1 Kcal/g diet, because higher levels, independent of the phytoestrogen content, can influence uterine weight (Odum et al. 2004; Thigpen et al. 2002) and time of VO.

The rationale for using the ovariectomized female rat or mouse in the uterotrophic bioassay is to reduce the levels of endogenous estrogens to an absolute minimum and to increase the sensitivity and reliability of the uterotrophic assay. It is therefore difficult to understand why similar efforts to minimize exogenous sources of estrogenicity in the diet have not been adopted. Eliminating dietary sources of phytoestrogens as much as possible and using a “sensitive” rat or mouse strain when conducting the uterotrophic bioassay would serve to increase the accuracy, sensitivity, and reproducibility of the assay. Additionally, this would greatly increase the ability of researchers to compare studies across time and within or between laboratories.

### Future considerations

**Figure 10:**
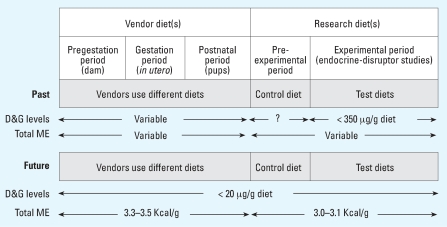
Future dietary considerations. Currently most experimental animals are fed at least two different diets containing variable levels of phytoestrogens and ME. Diet(s) should contain higher levels of ME for gestation and growth and lower levels of ME for VO and uterotrophic assays. Animal vendors should give serious consideration to providing animals that have been maintained on diets free of D&G for studies that can be affected by dietary estrogens.

Currently, most experimental animals are fed a range of diets with variable concentrations of phytoestrogens and energy levels. When purchasing rodents from different animal vendors, the investigator has little or no knowledge of the diet or the concentration of phytoestrogen in the diet used during gestation, weaning, and prior to delivery of the animals to the research laboratory (Figure 10). Studies indicate that exposure early in life and during gestation to levels of phytoestrogens typically found in most commercial rodent diets alters the sensitivity of rodents to carcinogens (Cotroneo et al. 2001; Lamartiniere et al. 2002) and also influences gene expression and phenotype (Dolinoy et al. 2006; Naciff et al. 2004; Wang et al. 2005). One example of this is the Agouti mouse in which exposure to genistein during pregnancy leads to changes in gene expression that alters coat color of the offspring. Eliminating sources of phytoestrogens in the diet fed to animals during gestation and in the pre- and post weaning periods would lead to greater consistency in experimental designs. We recognize that removing significant sources of phytoestrogens from the usual diet for some animal models may significantly influence biochemical, molecular, and genetic markers, leading to a changed phenotype that more closely resembles that seen before soy meal was used to formulate rodent diets. This will result in a need to reestablish baseline characteristics of the animal model, and for the VO end points or the uterotrophic bioassay it will undoubtedly improve the sensitivity of the assay. Currently, most manufacturers of rodent diets have commercially available diets that are formulated by omitting soybean meal and alfalfa meal, and thus contain only trace levels of phytoestrogen. In the future, animal vendors rearing and supplying animals should consider using diets that contain only trace levels of phytoestrogens, especially for use in estrogenic studies or for studies that measure estrogen-responsive elements as the end points. For testing of EDCs using the uterotrophic bioassay, it is logical to eliminate, as much as possible, known sources of phytoestrogens from the diet. Furthermore, to maximize the sensitivity of the assay, consideration should be given to the use of animal species or strains that are the most sensitive to dietary estrogenic substances. In this regard, we have clearly shown that the S-D rat is not the most sensitive rodent for such testing.

## Conclusion

We conclude that *a*) the CD-1 mouse and the F344 rat are considerably more sensitive to dietary phytoestrogens than the S-D rat; *b*) the same diet milled on different dates may have significantly different phytoestrogen content, which will cause marked differences in the time of VO in CD-1 mice and F344 rats, but less so in S-D rats; *c*) variations in the phytoestrogen content of rodent diets could be overcome by using soy- and alfalfa-free diets and by eliminating other known sources of estrogens; and *d*) a standardized open-formula phytoestrogen-deficient diet (one essentially devoid of known phytoestrogens) providing approximately 3.1 Kcal/g diet of ME, coupled with a more estrogen-sensitive rodent model (e.g., CD-1 mice, F344 rats) would be most appropriate to use in bioassays for evaluating the estrogenic activity of EDCs.

## Figures and Tables

**Table 1:** Body weights of S-D rats and F344 rats (mean ± SD) fed the PMI 5K96 control diet, different mill dates of the PMI 5002 diet containing different levels of D&G (98, 223, or 431 μg/g diet), or the AIN-76A diet spiked with genistein (μg/g diet).

		Body weight (g)			
Rat strain	Phytoestrogen (μg/g diet)	PND19	PND26	PND33	PND40
	D&G				
S-D	5K96 (< 7)	48.8 ± 4.65	82.2 ± 7.39	123.2 ± 9.68	167.8 ± 13.04
	5002 (98)	48.0 ± 5.50	84.8 ± 7.45	125.2 ± 10.60	169.1 ± 13.46
	5002 (223)	50.0 ± 4.64	85.6 ± 6.76	128.5 ± 10.46	169.0 ± 10.35
	5002 (431)	47.6 ± 5.15	82.3 ± 9.54	123.3 ± 13.28	163.2 ± 18.27
F344^a^	5K96 (< 7)	26.1 ± 1.70	56.1 ± 4.49	ND	101.6 ± 3.53
	5002 (98)	24.7 ± 2.21	52.5 ± 5.38	ND	99.2 ± 6.89
	5002 (223)	25.1 ± 1.99	54.9 ± 5.84	ND	99.3 ± 5.17
	5002 (431)	26.8 ± 1.56	56.6 ± 4.89	ND	100.4 ± 5.20
	Genistein				
S-D	AIN-76A				
	(0)	50.0 ± 4.87	85.8 ± 6.93	128.9 ± 8.20	174.7 ± 4.34
	(150)	48.2 ± 3.75	83.6 ± 6.06	123.6 ± 8.59	169.0 ± 3.49
	(300)	50.2 ± 4.21	86.1 ± 5.39	123.3 ± 10.14	166.3 ± 6.58
	(450)	47.6 ± 3.71	81.8 ± 6.68	122.9 ± 8.16	167.1 ± 0.27
F344	AIN-76A				
	(0)	27.9 ± 2.74	51.8 ± 4.15	77.1 ± 4.71	95.9 ± 5.79
	(150)	27.0 ± 3.50	51.3 ± 5.30	76.6 ± 6.31	94.2 ± 7.62
	(300)	26.5 ± 1.68	51.0 ± 2.84	75.3 ± 4.06	92.1 ± 5.64
	(450)	26.1 ± 1.75	48.5 ± 2.79	73.5 ± 3.41	94.4 ± 4.32

ND, not determined. *n* = 8 to 20 rats/group, 4 rats/cage.

a Body weights for F344 rats fed different mill dates of the PMI 5002 or PMI 5K96 control diet were recorded on PNDs 19, 29, and 40.

**Table 2:** Body weight, feed consumption, and estimated dose levels of D&G consumed by S-D rats fed the PMI 5K96 control diet or different mill dates of the PMI 5002 diet containing different levels of D&G (98, 223, or 431 μg/g diet).

Characteristic/diet	PNDs 19–26	PNDs 26–33	PNDs 33–40
Body weight (g)^a^			
5K96	65.5 ± 5.91	102.7 ± 8.46	145.7 ± 11.29
5002 (98)	66.4 ± 5.12	105.0 ± 7.89	147.7 ± 12.12
5002 (223)	67.8 ± 5.49	107.0 ± 8.44	141.6 ± 11.88
5002 (431)	65.0 ± 7.17	102.8 ± 10.93	141.5 ± 16.19
Feed consumption/rat/day (g)^b^			
5K96 (7)	10.0 ± 0.45	15.0 ± 0.76	17.3 ± 0.68
5002 (98)	9.4 + 0.26	13.2 ± 1.56	16.3 ± 0.70
5002 (223)	9.4 ± 0.43	14.2 ± 0.58	17.5 ± 1.72
5002 (431)	9.0 ± 0.51	16.0 ± 0.80	17.3 ± 1.49
Estimated dose (mg/kg/day)^c^			
5002 (98)	13.9 ± 0.39	12.4 ± 1.41	10.8 ± 0.23
5002 (223)	30.8 ± 0.80	29.7 ± 0.66	26.6 ± 2.96
5002 (431)	59.9 ± 1.94	67.2 ± 4.40	52.7 ± 2.34

a Mean ± SD for days in range (*n* = 8 to 20 rats/group).

b Calculated for each rat based on the weekly consumption per cage (4 rats/cage).

c Calculated based on the average weekly body weight from PNDs 19–26, 26–33, and 33–40 and the average feed consumed per rat per day based on the total consumed by the cage.

**Table 3:** The effects of the AIN-76A diet containing 0, 150, 300, or 450 μg genistein/g diet on the timing of VO and body weights (g) in CD-1 mice.

Genistein (μg/g diet)	Weaning weight (g)	PND of VO	Weight at VO (g)	Weight gain, PND15 to VO (g)	PND22 weight (g)	Sac weight (g)	Weight gain PNDs 15–22 (g)	Weight gain PNDs 15–30 (g)
0	8.76 ± 0.80	25.4 ± 2.69	17.61 ± 1.80	8.85 ± 2.31	14.29 ± 1.15	20.28 ± 1.28	5.53 ± 0.65	11.52 ± 1.40
150	8.61 ± 0.86	22.93 ± 2.60**	15.60 ± 2.22**	6.99 ± 2.43*	14.65 ± 1.33	20.35 ± 1.60	6.03 ± 1.06	11.74 ± 1.65
300	8.41 ± 0.88	20.97 ± 1.13#	12.95 ± 1.05#	3.82 ± 0.60#	13.82 ± 1.34	20.93 ± 1.52	5.41 ± 0.83	12.53 ± 1.59
450	8.42 ± 1.38	19.80 ± 0.84#	12.24 ± 1.32#	3.82 ± 0.60#	14.16 ± 1.98	21.06 ± 2.36	5.74 ± 0.94	12.56 ± 1.41

Mice were weaned on PND15 and sacrificed (sac) on PND30. Values shown are mean ± SD. *n* = 15 per group except for the 450-μg/g diet group, where *n* = 14 for measurements taken after PND22 [i.e., sac weight, weight gain PNDs 15–30; these values were not statistically significant (*p* > 0.05)]. *p*-Values reflect the significance of the linear trend. PND of VO is inversely related to the dose of genistein. Regression model: PND of VO = 25.1 – 0.0126 × dose; *R*^2^ = 53%; Pearson’s correlation = –0.73.

* *p* < 0.05

** *p* < 0.01, and

# *p* < 0.001 compared with control group.

**Table 4:** Comparative body weight, feed consumption, and estimated dose levels of genistein consumed by S-D and F344 rats exposed to the AIN-76A diet containing 0, 150, 300, or 450 μg genistein/g diet from PND19 to PND40.

	S-D rats			F344 rats		
Characteristic/diet	PNDs 19–26	PNDs 26–33	PNDs 33–40	PNDs 19–26	PNDs 26–33	PNDs 33–40
Body weight (g)^a^						
0	67.9 ± 5.49	107.3 ± 7.34	140.9 ± 30.79	39.9 ± 3.40	64.4 ± 4.35	86.5 ± 5.20
150	65.9 ± 4.62	103.3 ± 7.27	146.4 ± 10.32	39.3 ± 4.25	63.9 ± 5.77	85.6 ± 7.27
300	68.2 ± 3.59	104.5 ± 5.69	144.0 ± 15.25	38.8 ± 2.05	63.1 ± 3.13	83.0 ± 5.11
450	64.7 ± 4.75	101.6 ± 7.01	144.9 ± 12.13	37.3 ± 2.00	61.0 ± 2.75	84.1 ± 3.70
Feed consumption/rat/day (g)^b^						
0	7.6 ± 0.37	11.4 ± 0.35	13.9 ± 0.68	5.7 ± 0.18	6.5 ± 0.09	8.3 ± 0.12
150	7.3 ± 0.24	11.2 ± 0.49	13.5 ± 0.36	5.2 ± 0.54	6.2 ± 0.83	7.9 ± 0.84
300	7.5 ± 0.26	11.1 ± 0.56	13.4 ± 0.32	5.7 ± 0.15	6.5 ± 0.26	8.0 ± 0.38
450	7.3 ± 0.72	11.0 ± 0.88	13.7 ± 0.09	5.4 ± 0.13	6.4 ± 0.26	8.3 ± 0.30
Estimated dose (mg/kg/day)^c^						
150	16.8 ± 1.25*	16.3 ± 1.27*	13.9 ± 0.98	20.0 ± 2.46*	14.9 ± 1.88*	14.1 ± 1.66
300	33.2 ± 1.89*	32.8 ± 1.89*	28.2 ± 3.29	43.9 ± 2.38*	30.8 ± 1.65*	29.8 ± 2.07
450	49.9 ± 5.18*	50.2 ± 5.25	44.6 ± 2.68	64.9 ± 3.19*	47.0 ± 2.11	44.9 ± 1.93

a Mean ± SD for days in range (*n* = 8 to 20 rats/group).

b Calculated for each rat based on the weekly consumption per cage (4 rats/cage).

c Calculated based on the average weekly body weight from PNDs 19–26, 26–33, and 33–40 and the average feed consumed per rat per day based on the total consumed by the cage.

* Significantly different between S-D and F344 rats at *p* < 0.05 by two-sample *t*-tests.

**Table 5:** Study III: comparative effects of D&G and total ME on the timing of VO in F344 rats.

Test diet	Fat (%)	Protein (%)	ME (Kcal/g)	D&G (μg/g diet)	No.	VO (mean ± SD)	Significantly (*p* < 0.01) greater than diets
Group I: low phytoestrogen diets							
1. Harlan Teklad 2014S	3.5	14	3.10	5	16	42.1 ± 4.01	All other
2. Harlan Teklad 2016S	3.5	16	3.20	0	20	38.7 ± 4.01	All but 1 and 9
3. Harlan Teklad 2019	9.0	19	3.43	5	16	34.8 ± 2.54	7 and 12
8. Ziegler 5412–01	4.0	18	3.15	0	20	35.8 ± 2.84	7 and 12
9. PMI 5K96	4.0	19	3.15	0	32	38.2 ± 2.59	All but 1 and 2
11. AIN-76A	5.0	20	3.83	0	16	33.6 ± 1.55	None
Group II: medium phytoestrogen diets							
5. PMI 5002 (B3)	4.5	20	3.10	98	20	36.0 ± 2.49	7 and 12
12. AIN-76A soy	5.0	20	3.79	169	16	32.3 ± 1.25	None
Group III: high phytoestrogen diets							
4. PMI 5001	4.5	23	3.04	285	12	36.2 ± 1.03	None
6. PMI 5002 (B2)	4.5	20	3.10	223	20	33.9 ± 1.54	None
7. PMI 5002 (B1)	4.5	20	3.10	431	20	32.6 ± 1.54	None
10. Harlan Teklad 8656	4.0	24	2.96	347	16	33.0 ± 0.63	None

Data are results from two experiments: experiment 1 included diets 2, 4, 5, 8, and 9, and experiment II included all diets but diet 4. VO times were significantly (*p* < 0.01) greater in experiment I than in experiment II; although the summary statistics combine the two experiments, the significant study-to-study variability was taken into account in the statistical analysis. The most important factor related to VO for these data is phytoestrogen level, although ME is also significantly (*p* < 0.01) associated with VO time. Both variables show a negative correlation with VO (i.e., the later VO times are associated with lower values of ME and lower levels of phytoestrogens).

**Table 6:** Comparative mean time (± SD) of VO in F344 vs. S-D rats fed the 5K96 diet or diets with variable levels of D&G (98, 223, or 431 μg/g diet) and ME.

Rat strain	Low ME diet	D&G (μg/g diet)	Mean PND	High ME diet	Genistein (μg/g diet)	Mean PND	Difference in time of VO due to ME
F344	5K96	< 7	38.1 ± 2.8	AIN-76A	0	36.8 ± 2.8	1.3–3.2^a^
	5002	98	35.5 ± 3.8		150	35.2 ± 3.4	
	5002	223	33.9 ± 1.5		300	34.0 ± 2.3	
	5002	431	32.6 ± 1.5		450	26.8 ± 4.2	
S-D	5K96	< 7	31.9 ± 1.9	AIN-76A	0	29.6 ± 2.2	2.3
	5002	98	32.5 ± 1.4		150	30.3 ± 2.2	
	5002	223	32.7 ± 1.9		300	30.2 ± 2.2	
	5002	431	32.7 ± 1.9		450	27.1 ± 2.4	
Difference due to rat strain (days)			6.2			7.2	
Difference due to phytoestrogens							
F344			5.5			10.0	
S-D			0.8			3.2	

Low ME = 3.15 Kcal/g (5K96) or 3.10 Kcal/g (5002); high ME = 3.83 Kcal/g (AIN-76A).

a F344 rats fed AIN-76A phytoestrogen-free diet shown in Table 5.
